# A novel deconvolution method for modeling UDP-*N*-acetyl-D-glucosamine biosynthetic pathways based on ^13^C mass isotopologue profiles under non-steady-state conditions

**DOI:** 10.1186/1741-7007-9-37

**Published:** 2011-05-31

**Authors:** Hunter NB Moseley, Andrew N Lane, Alex C Belshoff, Richard M Higashi, Teresa WM Fan

**Affiliations:** 1 Department of Chemistry and Center for Regulatory & Environmental Analytical Metabolomics (CREAM), University of Louisville, Louisville, KY 40292, USA; 2 Structural Biology Program, JG Brown Cancer Center, University of Louisville, Louisville, KY 40292, USA; 3 Department of Medicine, Clinical Translational Research Building, Louisville KY 40202, USA; 4 Department of Pharmacology and Toxicology, University of Louisville, Louisville, KY 40202, USA

## Abstract

**Background:**

Stable isotope tracing is a powerful technique for following the fate of individual atoms through metabolic pathways. Measuring isotopic enrichment in metabolites provides quantitative insights into the biosynthetic network and enables flux analysis as a function of external perturbations. NMR and mass spectrometry are the techniques of choice for global profiling of stable isotope labeling patterns in cellular metabolites. However, meaningful biochemical interpretation of the labeling data requires both quantitative analysis and complex modeling. Here, we demonstrate a novel approach that involved acquiring and modeling the timecourses of ^13^C isotopologue data for UDP-*N*-acetyl-D-glucosamine (UDP-GlcNAc) synthesized from [U-^13^C]-glucose in human prostate cancer LnCaP-LN3 cells. UDP-GlcNAc is an activated building block for protein glycosylation, which is an important regulatory mechanism in the development of many prominent human diseases including cancer and diabetes.

**Results:**

We utilized a stable isotope resolved metabolomics (SIRM) approach to determine the timecourse of ^13^C incorporation from [U-^13^C]-glucose into UDP-GlcNAc in LnCaP-LN3 cells. ^13^C Positional isotopomers and isotopologues of UDP-GlcNAc were determined by high resolution NMR and Fourier transform-ion cyclotron resonance-mass spectrometry. A novel simulated annealing/genetic algorithm, called 'Genetic Algorithm for Isotopologues in Metabolic Systems' (GAIMS) was developed to find the optimal solutions to a set of simultaneous equations that represent the isotopologue compositions, which is a mixture of isotopomer species. The best model was selected based on information theory. The output comprises the timecourse of the individual labeled species, which was deconvoluted into labeled metabolic units, namely glucose, ribose, acetyl and uracil. The performance of the algorithm was demonstrated by validating the computed fractional ^13^C enrichment in these subunits against experimental data. The reproducibility and robustness of the deconvolution were verified by replicate experiments, extensive statistical analyses, and cross-validation against NMR data.

**Conclusions:**

This computational approach revealed the relative fluxes through the different biosynthetic pathways of UDP-GlcNAc, which comprises simultaneous sequential and parallel reactions, providing new insight into the regulation of UDP-GlcNAc levels and *O*-linked protein glycosylation. This is the first such analysis of UDP-GlcNAc dynamics, and the approach is generally applicable to other complex metabolites comprising distinct metabolic subunits, where sufficient numbers of isotopologues can be unambiguously resolved and accurately measured.

## Background

Stable isotope tracing is a powerful technique for delineating metabolic pathways and fluxes in response to external perturbations in a wide variety of systems [1-4]. We have been developing stable isotope-resolved metabolomic analysis (SIRM) for polar and non-polar metabolites in cell and tissue systems to obtain a comprehensive view of the flow of carbon or nitrogen through different metabolic pathways [5-12].

**Figure 1:**
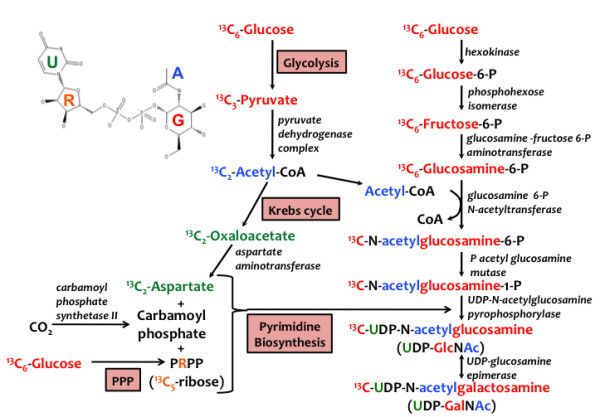
**Biosynthesis of UDP-*N*-acetyl-D-glucosamine (UDP-GlcNAc)**. The pathways from [U-^13^C]-glucose to the four biochemical subunits are outlined. The glucose moiety (red) is directly incorporated into UDP-GlcNAc. The acetyl moiety (blue) is incorporated via glycolysis. The ribose moiety (yellow) is incorporated via the pentose phosphate pathway and pyrimidine biosynthesis. The uracil moiety is derived from acetyl-CoA through the Krebs cycle to form aspartate where it is combined with carbamoyl phosphate leading to pyrimidine synthesis (green).

This approach involves the combined use of NMR and mass spectrometry (MS), both at very high resolution, which respectively provide direct information on positional isotopomers and isotopologues (sometimes termed 'mass isotopomers') of labeled metabolites in an unfractionated mixture, thereby minimizing errors from sample processing [10]. NMR and MS are complementary structural techniques; both types of analytical information are crucial for accurate reconstruction of metabolic pathways leading to the synthesis of labeled metabolites [8,13]. For complex metabolites that are composed of several metabolic subunits such as UDP-*N*-acetyl-D-glucosamine (UDP-GlcNAc; see Figure 1), the observed isotopologues are mixtures of several positional isotopomers even at the level of the individual metabolic subunits. This degeneracy makes pathway reconstruction and metabolic flux modeling extremely complex. To solve this problem we have developed a technique for deconvoluting ^13^C isotopologues into all possible labeled subunits with ^13^C distribution in the subunits mapped based on knowledge of their biosynthetic pathways. This approach is illustrated here with the metabolite UDP-GlcNAc, which is composed of four metabolic modules, namely uracil (U), ribose (R), glucose (G) and acetyl (A) (Figure 1). The technique can be applied to any complex metabolite where a sufficient number of mass isotopologues can be identified and accurately quantified.

UDP-GlcNAc is an activated precursor for both *N*-linked and *O*-linked glycosylation of proteins, which are important in regulating numerous cellular processes, such as protein targeting to organelles [14] and nutrient sensing [15,16]. These two major glycosylation pathways in eukaryotic cells differ in the protein targets and cellular localization [17]. With *O*-linked glycosylation, cytoplasmic and nuclear proteins are modified by the transfer of a single β-*N*-acetylglucosamine (GlcNAc) unit from UDP-GlcNAc to the oxygen of Ser or Thr side chains of proteins. This reaction is catalyzed by the enzyme uridine diphospho-*N*-acetylglucosamine:polypeptide β-*N*-acetylglucosaminyltransferase (*O*-GlcNAc transferase, or OGT). *O*-Linked GlcNAcylation has been shown to participate in a variety of cytoplasmic and nuclear regulatory processes in response to stress in a fashion both similar and complementary to phosphorylation [15,18-20]. *O*-GlcNAc modified proteins including the polycomb group, p53, c-Myc, insulin receptor have been linked to the regulation of embryonic development [21], cancer [22,23] and diabetes [24]. In addition, the synthesis and turnover of these modified proteins are tightly regulated, which implies that the supply of the precursor UDP-GlcNAc must also be tightly regulated.

Using ultra-high-resolution and accurate mass Fourier transform-ion cyclotron resonance-MS (FT-ICR-MS) and high-resolution NMR, we have identified four major sugar nucleotides including UDP-Glc and UDP-GlcNAc directly in crude extracts of mammalian cells. The biosynthesis of UDP-GlcNAc is complex as it involves the interplay of both sequential and parallel metabolic pathways (see Figure 1). Thus, one must simultaneously consider glycolysis, the hexosamine biosynthetic pathway (HBP), the Krebs cycle, the pentose phosphate pathway, and the pyrimidine biosynthetic pathway when investigating UDP-GlcNAc metabolism. Fortunately, isotopomer distributions in several key metabolites ('reporters') of these pathways, including lactate, glucose, UDP-GlcNAc, and uridine, can be readily identified and quantified by NMR [10,25]. For abundant or sufficiently enriched metabolites, NMR is also excellently suited for following the time evolution of positional isotopomers in cell culture and *in vivo *([4,26-28], and see below). For less abundant metabolites and where isotopic steady state is difficult to achieve, such as in mammalian cell cultures, the more sensitive FT-ICR-MS technique is advantageous. However, mass spectrometry measures isotopologues, which must be deconvoluted into individual isotopomer species for dynamic flux analysis.

For flux analysis, detailed times courses are also needed for systems that are not in isotopic steady state. Numerous modeling techniques, including metabolic balance analysis [29-31], metabolic control analysis [31-33] have been developed which use a series of differential equations to model the flux of metabolites. These techniques typically require steady-state conditions that apply standard numerical methods to solve a system of differential equations in the form of an eigensystem, though there are a few techniques that can be applied to non-steady-state conditions [34,35]. While steady-state conditions are often assumed, in reality they are difficult to establish, maintain, and verify for all relevant metabolites in experiments involving mammalian cells. Most of these modeling techniques rely on total metabolite concentrations or isotopic enrichment ratios of a limited number of metabolites, which creates an underdetermined system of equations where there are more variables than independent data. Thus, unique meaningful solutions to these numerical systems are not always practical [36].

**Figure 2:**
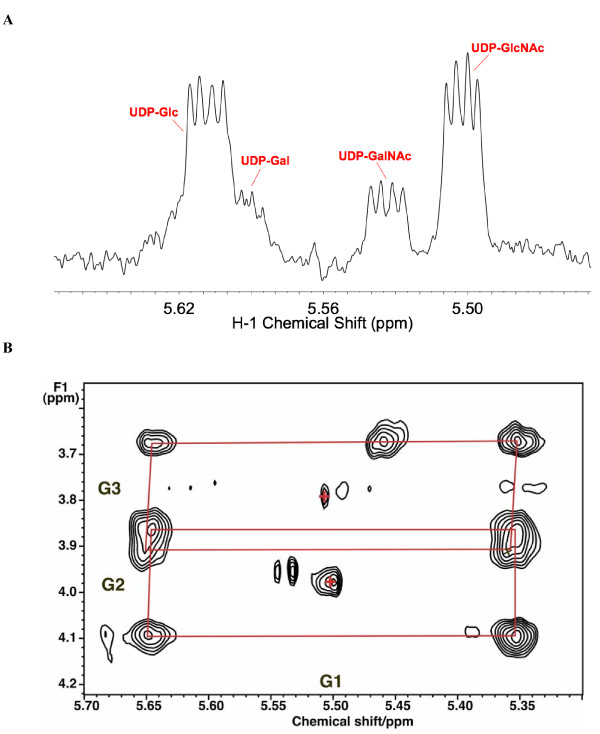
**Species assignments of UDP-*N*-acetyl-D-glucosamine (UDP-GlcNAc) isotopologues in Fourier transform-ion cyclotron resonance-mass spectrometry (FT-ICR-MS)**. The same crude extracts used for NMR were analyzed following re-exchange of ^2^H back to ^1^H. Analysis conditions are stated in the text. With correction to an internal reference, all of the isotopologues were assignable at better than 1 ppm mass accuracy, with most better than 10 ppb mass accuracy. The molecular formulae were assigned using Xcalibur software with elemental limits set to CHONP and allowing up to 17 occurrences of ^13^C. The combination of the ultra-high resolution with extreme mass accuracy resulted in high confidence that only 'pure' ^13^C isotopologues were quantified for the moiety modeling.

Here, we have used both NMR and FT-ICR-MS to probe the biosynthesis of UDP-GlcNAc in prostate cancer cells, coupled with the development of algorithms to deconvolute the resulting MS data. LnCaP-LN3 prostate cancer cells were grown in [U-^13^C]-glucose to trace the time-dependent fate of individual ^13^C atoms in different metabolic subunits of UDP-GlcNAc. The ^13^C isotopologue distribution in UDP-GlcNAc was measured by direct infusion nanoelectrospray FT-ICR-MS. As we have previously shown, the resolution in the MS is sufficient to resolve all 17 ^13^C isotopologues of UDP-GlcNAc (Figure 2) from other metabolites and other elemental isotopologues (for example, ^2^H, ^15^N, ^18^O, and all possible combinations of isotopes) of UDP-GlcNAc plus providing intensity measurements with an accuracy and precision of better than 1% [5]. The MS data was then deconvoluted and modeled using the newly developed algorithm. This combined analytical and computational approach generated much more independent isotopomer data to help minimize the problem under determination. To the best of our knowledge, this is the first detailed kinetic analysis of the UDP-GlcNAc pathways by stable isotope analysis.

## Results

### Metabolism of LN3 cells

**Figure 3:**
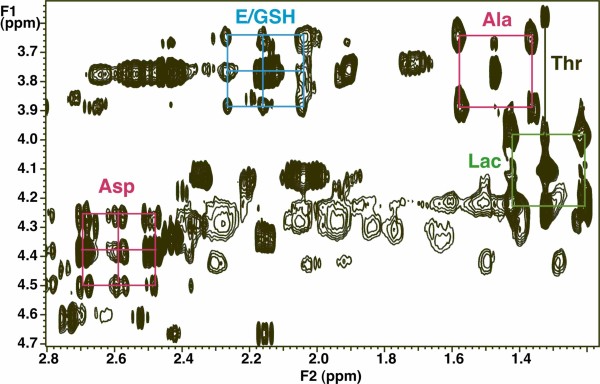
**13C Labeling in metabolites that report on glycolysis, Krebs cycle and uracil biosynthesis**. The two-dimensional ^1^H total correlation spectroscopy (TOCSY) spectrum was recorded at 18.8 T using an isotropic mixing time of 50 ms at a B_1 _field strength of 8 kHz. The ^13^C satellites of glutamate and glutamate in reduced glutathione C2H-C4H are shown in cyan. This pattern corresponds to a mixture of species, namely where both ^13^C atoms are labeled in the same molecule plus ^13^C2^12^C4 and ^12^C2^13^C4. In contrast, the patterns for Ala (red) and Lac (green) shows only the ^13^C3^13^C2 plus ^12^C3^12^C2 pattern. Aspartate (red) shows a similar pattern as glutamate, reflecting scrambling through the Krebs cycle [8,10,11], and is the same as in U in UTP.

**Figure 4:**
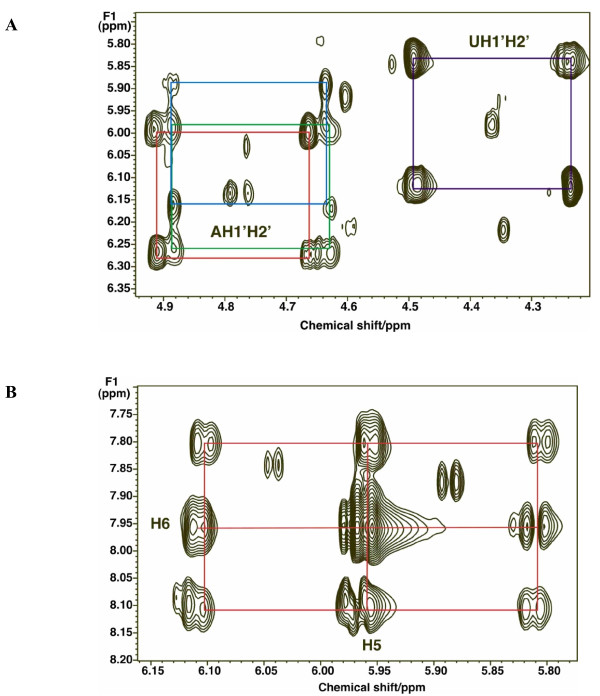
**Two-dimensional ^1^H total correlation spectroscopy (TOCSY) spectra of components of UDP-*N*-acetyl-D-glucosamine (UDP-GlcNAc)**. (a) Ribose ring region of the TOCSY spectrum (see Figure 3). The ribose moieties of the free nucleotides are essentially completely labeled by 48 h. Adenine nucleotides AXP H1'-H2' (red); uracil nucleotides UXP H1'-H2' (purple). (b) Uracil ring in UXP shows the scrambled pattern in the C5-C6 positions of U as in the precursor aspartate residue (Figure 3).

**Figure 5:**
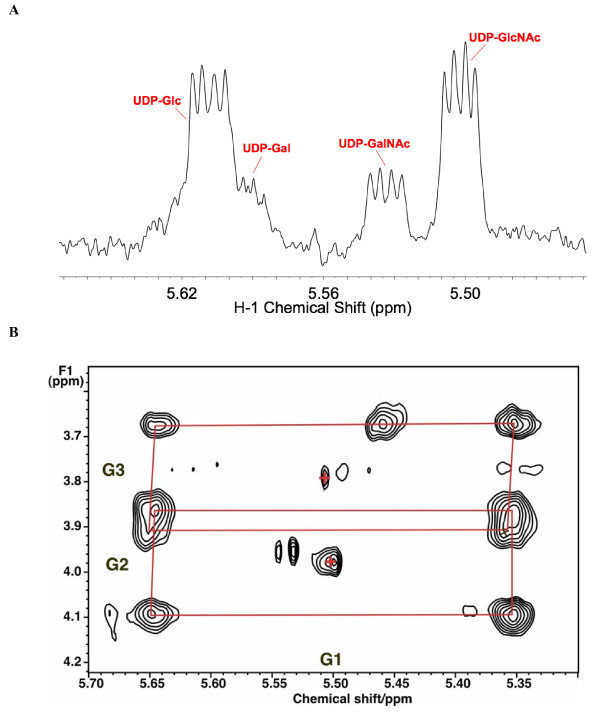
**NMR identification of UDP-hexoses**. LN3 cells were grown in unlabeled glucose and extracted as described in the Methods section. One-dimensional ^1^H NMR spectra were recorded at 20°C, 800 MHz. Two-dimensional ^1^H total correlation spectroscopy (TOCSY) spectra were recorded at 600 MHz using a mixing time of 50 ms with a spin lock field strength of 8 kHz. **(a) **One-dimensional NMR spectrum. The sugar anomeric region shows several resonances that are double doublets, that is, a single proton scalar coupled to two different spin 1/2 nuclei. By comparison with standard spectra, these have been assigned to the glucose H1 of UDP-glucose, UDP-galactose (UDP-Gal), UDP-GalNAc and UDP-GlcNAc by ^1^H and ^13^C chemical shifts, splitting patterns, ^13^C labeling and two-dimensional TOCSY crosspeak patterns as described in the text. **(b) **Anomeric region of example TOCSY spectrum showing ^13^C satellites of glucose H1-H3 of UDP-GlcNAc due to incorporation of [U-^13^C]-glucose. The satellite crosspeaks (denoted by red rectangles) represent the covalent linkages of H1 to H2 and H3 of the glucose moiety (labeled respectively as G1, G2 and G3), which were essentially completely labeled at 48 h. Crosses denote the crosspeaks of protons attached to ^12^C.

As with many other cancer cells in culture, ^13^C glucose is a major source of carbon for nucleotide riboses (via the pentose phosphate pathways), amino acids such as Ala, Glu and Asp via glycolysis and the Krebs cycle, and pyrimidine rings in LnCaP-LN3 cells. These metabolites are in turn precursors of glutathione, proteins, pyrimidine nucleotides and sugar nucleotides. The unlabeled and labeled metabolite profiles of the LN3 cells and media grown in the presence of [U-^13^C]-glucose were determined by NMR and mass spectrometry as described in the Methods section. We have determined the relative rates of consumption of glucose, glutamine and essential amino acid, and lactate production in the medium by NMR (Additional file 1). As the number of cells approximately doubled during the measurement period, the determination of the rates of consumption per unit of cells is complicated, and leads to inconstant rates over time, as we have previously discussed [37]. The initial rates per mg dry weight are given in Additional file 1. Under the conditions used (initial 1.4 million cells), the rate of glutamine consumption was sevenfold slower than the glucose uptake rate. From the rate of ^13^C lactate excretion, we calculated that about 38% of the consumed glucose was converted to lactate and excreted, which is comparable to other cancer cells in culture [9,38]. Furthermore, around 30% of the glucose was consumed by 48 h (Additional file 1). Many other intracellular metabolites and their ^13^C isotopomers that report on central metabolic pathways were identified and quantified. These included the intracellular lactate and Ala, Glu, Asp and uracil (that report on glycolysis, Krebs cycle and pyrimidine biosynthesis), and the ribose components of the free nucleotide pools that reflect the pentose phosphate pathway (Figures 3 and 4 and Table 1). We also identified four sugar nucleotides (Figure 5a), which are associated with these carbon flows (see Figure 1).

**Table 1:** Quantification of ^13^C enrichments in relevant metabolites by ^1^H total correlation spectroscopy (TOCSY) at 48 h

Compound	Percentage ^13^C
Uracil in UXP	

^12^C6^12^C5	52 ± 3

^12^C6^13^C5	14 ± 2

^13^C6^12^C5	16 ± 2

^13^C6^13^C5	18 ± 2

Asp	

^12^C2^12^C3	46 ± 3

^12^C2^13^C3	15 ± 2

^13^C2^12^C3	19 ± 2

^13^C2^13^C3	21 ± 2

AXP ribose	

^12^C1'^12^C2'	8 ± 2

^13^C1'^13^C2'	92 ± 2

UXP ribose	

^12^C1'^12^C2'	4 ± 1

^13^C1'^13^C2'	96 ± 2

UDP GlcNAc	

Glc ^12^C1^12^C2	9 ± 2

Glc ^13^C1^13^C2	91 ± 2

Lactate	

^12^C2^12^C3	19 ± 2

^13^C2^13^C3	81 ± 2

Alanine	

^12^C2^12^C3	23 ± 2

^13^C2^13^C3	77 ± 2

Glutamate	

^12^C2^12^C4	63 ± 2

^12^C2^13^C4	32 ± 2

^13^C2^12^C4	< 1

^13^C2^13^C4	5 ± 2

^12^C2^12^C3	81 ± 2

^12^C2^13^C3	6 ± 2

^13^C2^12^C3	5 ± 2

^13^C2^13^C3	8 ± 2

Atom percentage ^13^C enrichments in metabolites after 48 h incubation in [U-^13^C]-glucose were determined from two-dimensional ^1^H TOCSY experiments as described in the Methods section. Uracil in UXP refers to the uracil ring in the free nucleotide pool comprising UTP, UDP and UMP. AXP and UXP ribose refer to the ribose moiety in the free nucleotide pools. UDP-*N*-acetyl-D-glucosamine (UDP-GlcNAc) refers to the C1-C2 of the glucose moiety of the free UDP-GlcNAc pool.

### Identification of sugar nucleotides

Figure 5a shows a partial ^1^H NMR spectrum that corresponds to the sugar nucleotide region. The resonance at 5.51 ppm shows the characteristic quartet pattern of the anomeric proton of a pyranose sugar unit in a nucleotide as a result of the three-bond coupling to H2 and ^31^P. The two-dimensional ^1^H total correlation spectroscopy (TOCSY) spectrum (Figure 5b) shows the expected pattern of ^13^C satellite crosspeaks for the glucosyl moiety of sugar nucleotides. Comparing the chemical shifts and scalar coupling patterns with those of authentic standards, we assigned these resonances to four different nucleotide sugars, namely UDP-Glc, UDP-GlcNAc, UDP-Gal and UDP-GalNAc. These were consistent with the molecular formula obtained from the ultra-high-mass resolution FT-ICR-MS (see below).

### Isotopomer distributions of metabolites determined by NMR

UDP-GlcNAc is composed of four metabolic units or modules, each of which is synthesized in different pathways (see Figure 1). The glucose unit derives directly from the supplied glucose without metabolic scrambling, as these cells are not known to be gluconeogenic. Similarly, the ribose can also be derived from the supplied glucose, or from the turnover of existing ribonucleotides. The uracil unit may be derived from RNA turnover, or *de novo *synthesis, which requires carbon input from aspartate and CO_2_. Aspartate can be produced by protein turnover or transamination of oxalacetate (OAA) (Figure 1). OAA can incorporate carbon from acetyl CoA, which is derived from glucose, or from glutamine carbon entering the Krebs cycle via glutaminolysis [4,38]. The acetyl moiety is derived either from glycolysis or fatty acid oxidation. The glucose pathways were readily discriminated from the non-glucose pathways by tracing the ^13^C label from [U-^13^C]-glucose into the various biosynthetic intermediates, as well as in UDP-GlcNAc itself (see Table 1).

After introduction of [U-^13^C]-glucose enriched medium, the free intracellular glucose was rapidly replaced by [U-^13^C]-glucose, and the metabolically proximal metabolites, such as the ribose rings of the free nucleotides also became highly labeled. The glycolysis markers, Ala and lactate were preferentially labeled from the glucose source, and the glucose unit within UDP-GlcNAc became > 90% enriched in ^13^C by 48 h. Based on the NMR data, these markers were either the all ^13^C, or the all ^12^C form, after correcting for natural abundance ^13^C at approximately 1.1%) (Figures 3, 4a and 5b; Table 1). In contrast, the downstream metabolites that report on both glycolysis and Krebs cycle activity (Asp, Glu) were considerably less ^13^C labeled, and also showed scrambling due to reactions with unlabeled intermediates in the Krebs cycle (for example, citrate synthase, 2-oxoglutarate dehydrogenase steps) (Figures 3 and 4b). A significant source of unlabeled Krebs cycle intermediates is glutamine (see above). As Asp is a direct precursor of uracil biosynthesis, we expected the final product would also show scrambled labeling patterns; the labeling pattern in Asp was essentially identical to that of U in UXP, as expected for a direct precursor-product relationship (see Table 1).

### Isotopologues of UDP-GlcNAc determined by FT-ICR-MS

**Figure 6:**
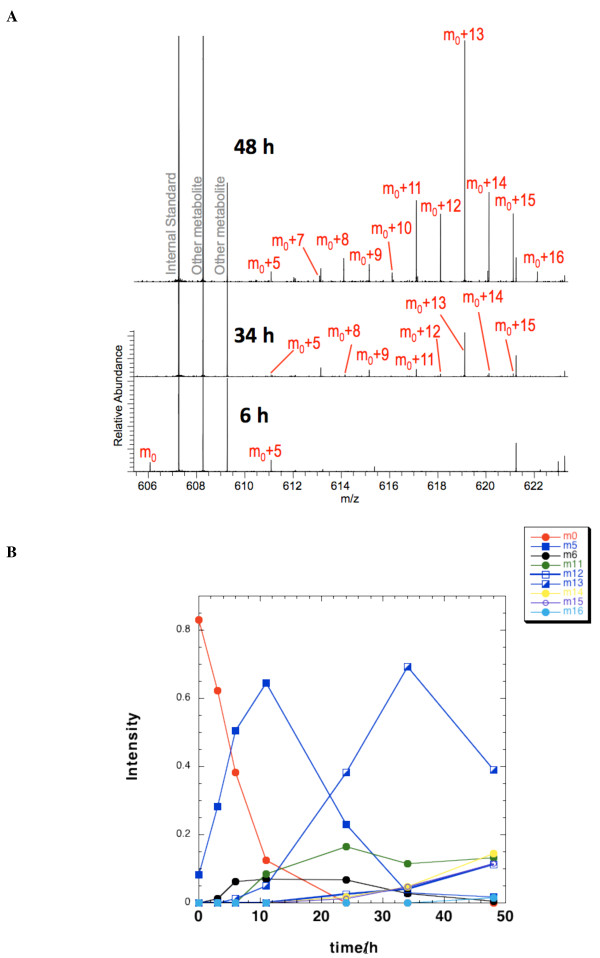
**Timecourse changes of UDP-*N*-acetyl-D-glucosamine (UDP-GlcNAc) isotopologues in LN3 extracts as determined by Fourier transform-ion cyclotron resonance-mass spectrometry (FT-ICR-MS)**. **(a) **FT-ICR-MS spectra at 6, 34 and 48 h post [U-^13^C]-glucose labeling. The spectra are plotted by normalization to cellular concentrations for direct comparison. For clarity, only the isotopologues visible at this scale are tagged; it should be noted that by 34 h the monoisotopic UDP-GlcNAc (m_0_) was barely detectable (data not shown). As is evident from this figure, different isotopologues increased in intensity at different rates, resulting in changing isotopologue distributions. M_0_+5, 7-16 correspond to the UDP-GlcNAc isotopologues with 5 and 7 to 16 ^13^C atoms, respectively. **(b) **Timecourses of intensity of selected mass isotopologues. The normalized intensities were obtained as described in the Methods section. Only mass isotopologues that reached a significant level are plotted. Symbols are defined in the figure. The lines only serve to connect the data points. M0, 5,6, 11-16 represent monoisotopic UDP-GlcNAc and isotopologues with 5, 6 and 11 to 16 ^13^C atoms, respectively.

The 17 possible ^13^C isotopologues of UDP-GlcNAc in LN3 cell extracts were analyzed using direct infusion nanoelectrospray FT-ICR-MS, which is especially well suited for mass isotopologue analysis. FT-ICR-MS requires no derivatization, the relative intensities are significantly more accurate than conventional MS, routine mass accuracy is better than 0.5 ppm, and the analysis is relatively high-throughput. Most importantly, the extremely high mass resolution makes interference from other metabolites and non-^13^C isotopologues highly unlikely [5]. Moreover, the high sensitivity and small sample requirements (low to sub-pmol levels of UDP-GlcNAc) make the technique well suited for timecourse measurements. The spectral assignments of the UDP-GlcNAc isotopologues in LN3 extracts are shown in Figure 6a, along with timecourse changes at 6, 34 and 48 h post labeling. The isotopologue peaks of UDP-GlcNAc are tagged as m_0 _to m_0_+16, which represent the monoisotopic (all ^12^C) to ^13^C_16 _species. The detailed timecourses of various isotopologue intensities are shown in Figure 6b, which display a typical precursor product relationship. Some isotopologues (that is, m_0_+3-4, m_0_+7-10) were not sufficiently enriched to determine the timecourse, and thus were not plotted. The m_0 _peak disappeared rapidly with a half life of 5.3 h (Figure 6b), reflecting replacement of the initial pool of unlabeled UDP-GlcNAc by newly synthesized ^13^C isotopologues. At zero time the fraction of the m_0 _peak was about 85%, close to the expected 83% from natural abundance. In contrast, the intensity of other isotopologues increased with time, and some took more than 1 d to become significantly labeled.

### Isotopologue analysis

Starting with ^13^C glucose, the complete synthesis of UDP-GlcNAc leads to a total of 17 isotopologues (see below), many of which comprise several isotopomers. However, as each isotopologue was independently quantified and the intensity profile contained more measurements than the number of independent isotopomer species, the observed profile can be deconvoluted into the individual isotopomer components in terms of biochemical units. The intensity at a given mass is the sum of the intensities of the individual isotopic species at that mass, and the net intensity due to ^13^C enrichment was corrected for natural abundance ^13^C contribution using the previously described stripping algorithm [39].

### Algorithm

UDP-GlcNAc comprises four biochemical units: glucose (G), ribose (R), acetyl (A) and  uracil (U); g0, r0, a0 and u0 represent the probability of finding unlabeled glucose, ribose, acetyl and uracil, respectively. The NMR data (Figures 2b and 4b) showed that the glucose and ribose units existed significantly only as fully ^13^C-labeled G6 and R5, or completely unlabeled (G0, R0) forms, after 24 and 48 h of labeling. Furthermore, the mass isotopologue timecourse (Figure 6a) showed absence of m_0 _to m_4 _intensity, which is consistent with these subunits being enriched with all ^13^C or none. However, it was unclear from the NMR data whether the possibility of isotopic mixing existed for the acetyl unit, which was specifically considered in the modeling (see below).

If g0 represents the mole fraction of ^12^C_6 _glucose, G0, it follows that:(1a)

and similarly for the ribose and acetyl units,(1b)

The biosynthesis of uracil mixes carbon from glucose and other sources such as glutamine through the Krebs cycle. This leads to several isotopologues, namely u0, u1, u2 and u3 where the indices 1, 2 and 3 refer to the number of ^13^C atoms in the ring, which can be measured by NMR (see below). Moreover, these probabilities sum to unity:(2)

Hence, there are 6 undetermined parameters to be calculated, and up to 32 possible isotopomers. However, at each timepoint there are 17 intensity measurements. If I_n _denotes the intensity of the nth isotopologue, then using Eq. 1 and 2:(3)

To solve these simultaneous equations (Eq. #3), we have developed a general simulated annealing/genetic algorithm for parameter optimization called Genetic Algorithm for Isotopologues in Metabolic Systems (GAIMS) [40], (available at http://bioinformatics.chem.louisville.edu/). This algorithm robustly finds the optimal solution for all intensities via fitting of these six independent parameters (that is, g0, r0, a0, u0, u1, and u2). The target function, T, which is minimized, is defined in Eq. (4) as:(4)

where, I_n,obs _and I_n,calc _are the observed and calculated intensities, respectively, at a given timepoint. This optimization method used a linear annealing regime along with a 5% crossover rate and a population size of 20. Furthermore, three variables were mutated per step to handle any issues of dependency between variables. Each optimization used 10^6 ^steps and was repeated 50 times to verify robustness (avoidance of local minima) and to provide statistics. In addition, GAIMS was applied to over 40 variant models that include the possibility of other isotopomers in the various subunits (see Additional file 1) and a robust model selection method using the Akaike information criterion (AIC) [41], which was applied to average optimized parameter values.

### Testing and implementation

The mole fractions of individual isotopomers were calculated from the isotopologue intensity distributions in Figure 6b using the six-parameter optimizations from the GAIMS algorithm as described in the Methods section. In addition, we used the Akaike information criteria (AIC)-based model selection method described in the Methods section to select among 40 variant models (see Additional file 1). The best model according to these criteria, which also made the most biological sense, was our original six-parameter model. Specifically, allowing for all isotopomers of the acetyl unit produced significantly worse AIC values [40], justifying our original approximation on all or none ^13^C labeling in the acetyl unit *post hoc*.

**Figure 7:**
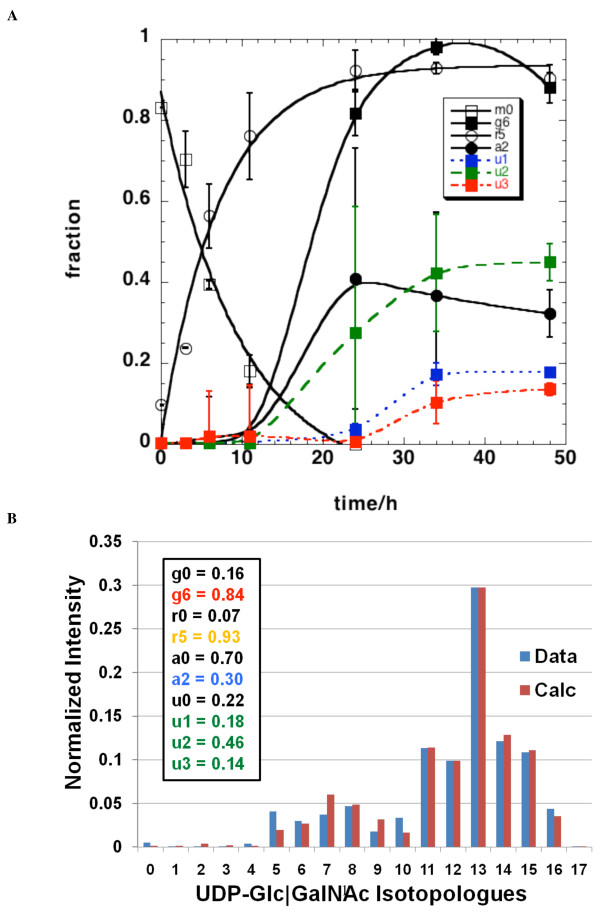
**Deconvolution of isotopologues of UDP-*N*-acetyl-D-glucosamine (UDP-GlcNAc) and kinetic modeling of their timecourses**. Mass isotopologues at each timepoint were deconvoluted into individual isotopomer components and their intensity was corrected for natural abundance contribution, as described in the Methods section. **(a) **Mole fractions of the various components are plotted with time. m_0 _(open square), ^13^C_6_-glucose g6 (black filled square),^13^C_5_-ribose r5 (open circle), ^13^C_2_-acetyl a2 (black filled circle), ^13^C_1_-uracil u1 (blue filled square),^13^C_2_-uracil u2 (green filled square), ^13^C_3_-uracil u3 (red filled square). The ribose component was fitted to the function b(1-exp-kt) with b = 0.89 and k = 0.13 h^-1 ^R^2 ^= 0.977. The unlabeled species (m_0_) was fitted to a single exponential decay I(t) = I(0)exp(-kt) with I(0) = 0.88 ± 0.06, k = 0.13 ± 0.02 h^-1 ^R^2 ^= 0.967. **(b) **Reconstruction of the isotopologue distribution from the mole fraction probabilities in A for the 48 h timepoint. The best fit values for the fractions were obtained according to equations 3,4 and 5 using the 'Genetic Algorithm for Isotopologues in Metabolic Systems' (GAIMS) as described in the text. The symbols are given on the figure. Blue bars are the observed intensities at each m/z and red bars are the reconstructed intensities.

Figure 7a shows the mole fraction of the deconvoluted isotopomer components (see the Methods section) as a function of time. The standard deviations were calculated from 50 independent optimizations, and were relatively small for most species except for the acetyl unit, which appeared to be less well determined. As Figure 7a indicates, the ^13^C enrichment into the ribose component was rapid (half life of approximately 6 h) and reached a plateau value of about 90%, similar to that measured by NMR (Table 1). The rate of ^13^C incorporation into the ribose component was similar to the rate of decrease in the unlabeled UDP-GlcNAc (Figure 7a), consistent with a direct precursor-product relationship. Interestingly, the enrichment into the glucose component showed a distinct lag of around 10 h, after which the enrichment reached a plateau value of > 90%, as evident also by NMR at 48 h. Furthermore, as the uracil and acetyl components require many more transformation steps, the enrichment into these species should take more time, as was observed (see Figure 7a).

The reconstruction of the isotopologue distribution from GAIMS modeling was compared with the observed values, as shown in Figure 7b for the 48 h timepoint. The agreement was as good as the variance in the data, and further modeling that included more species (such as ^13^C_1 _acetate) gave no improvement of the fitting, according to a variety of criteria (see the Methods section). Thus, based on the model calculation, the fraction of the uniformly ^13^C-labeled glucose, ribose, acetyl units in UDP-GlcNAc were respectively 0.84, 0.93, and 0.3, while the fraction of singly, doubly, and triply ^13^C-labeled uracil units was respectively 0.18, 0.46, and 0.14 at 48 h. These compare favorably with the independent isotopomer analysis by NMR at 48 h of incubation (see Table 1).

## Discussion

We have unequivocally identified four UDP-hexoses in LN3 cells using a combination of NMR and FT-ICR-MS data. In these cells, the most abundant sugar nucleotide was UDP-GlcNAc (Figure 1), whereas in other cancer or normal cells (for example, A549, MDAMB231, NHBE), UDP-Glc is the major sugar nucleotide (TW-M Fan and AN Lane, unpublished data). The abundance of UDP-GlcNAc in LN3 cells could mean a high synthesis rate, which could in turn drive a high OGT activity [42,43].

As Figure 1 shows, the biosynthesis of UDP-GlcNAc is complex, involving the coordination of glycolysis, Krebs cycle, pentose phosphate pathway, pyrimidine biosynthesis, and hexosamine biosynthesis. The total concentration of UDP-GlcNAc (and the other nucleotide hexoses) was maintained constant in LN3 cells over the timecourse, that is, at a steady state in which utilization was balanced by *de novo *synthesis. However, the ^13^C incorporation into the individual intermediates did not approach isotopic steady state for at least 30 h (Figure 7a). Thus, non-steady-state approaches [34,35,44] are required for the flux analysis.

The approach that we introduced here enabled a quantitative analysis of fractional contribution of relevant pathways to the synthesis of UDP-GlcNAc, regardless of whether the steady-state conditions are met. The UDP-GlcNAc molecule was dissected into four biochemical modules, each of which can utilize glucose as the carbon source. The exception is uracil, where one of the carbons comes from bicarbonate. In addition to glucose, alternative sources of carbon were also considered. The hexose unit in LN3 cells should derive exclusively from the supplied [U-^13^C]-glucose in the medium since there was no evidence for active gluconeogenesis in these cells. The ribose unit of the UTP pool was also derived mainly from [U-^13^C]-glucose. Acetyl CoA could be made from pyruvate via glycolysis, by fatty acid oxidation, or from glutaminolysis [45] by way of malic enzyme. For uracil, the C4, C5 and C6 carbons were derived from aspartate, which could be obtained from protein degradation or by the transamination of OAA. OAA could in turn come from [U-^13^C]-glucose via the Krebs cycle or amino acid oxidation, especially glutamine, which is a more direct carbon source than glucose. Our 6-parameter model based on the above rationalization of ^13^C incorporation from [U-^13^C]-glucose was corroborated by its comparison and preferred selection from over 40 variant models, representing many alternate pathways discussed above.

The resulting modeled timecourses for the various labeled intermediates (Figure 7a) are as expected for a largely sequential series of reactions. The isotopologue m_0_+5 represents the first labeled intermediate generated in the complex pathways (see also Figure 6b). All other significantly populated intermediates show clear lag phases as expected for sequential reactions that are effectively irreversible. It is notable that the length of the lag period increased as the number of ^13^C atoms increased (Figure 7a), which reflects the increasing number of reactions needed to achieve the labeling.

The decay of the fraction of the m_0 _species (I_0_) was quasi exponential (Figures 6b and 7a) and can be regarded as the rate of UDP-GlcNAc utilization (for example, incorporation into proteins). Since the total concentration of UDP-GlcNAc was constant, a decrease in the fraction of m_0 _was compensated by the synthesis of UDP-GlcNAc (that is, those bearing at least one ^13^C atom). The initial rate of formation of the m_0_+5 species was essentially equal to the rate of loss of m_0 _(Figures 6b and 7a), which corresponds to two possible isotopomer species (Equation 3). During the initial periods, the most likely species would be ^13^C_5_-ribose (fully labeled ribose, Figure 7a), as the acetyl CoA and uracil units take longer to become labeled and did not reach such a high degree of labeling (Figure 7a). Thereafter, the rapid rise and disappearance of the m_0_+5 isotopologue in Figure 6b indicates that initially the glucose, uracil and acetyl units of UDP-GlcNAc arose from pre-existing unlabeled sources, and that these sources are depleted relatively rapidly. Only then will *de novo *synthesis of UDP-GlcNAc have an increasing contribution from labeled glucose, uracil and acetyl CoA, leading to the fractional decrease of the m_0_+5 isotopologue.

The present timecourse was acquired by discrete sampling at separate timepoints, which could be done more elegantly by continuous *in vivo *NMR measurement [26,28]. However, UDP-GlcNAc is a relatively low abundance metabolite, which was difficult to detect by NMR. More importantly, the *in vivo *NMR analysis would lack the necessary resolving power to quantify as many isotopomer species of UDP-GlcNAc as required for the modeling effort. The superior sensitivity and resolution of the FT-ICR-MS techniques outweigh the *in vivo *NMR advantage and enabled modeling of the flux through the complex pathway, with much less ambiguity and under non-isotopic steady-state conditions.

## Conclusions

We have unequivocally identified four UDP-hexoses in LN3 cells of which the most abundant was UDP-GlcNAc using a combination of NMR and FT-ICR-MS data. The ^13^C incorporation into the individual intermediates did not approach isotopic steady state for at least 30 h, thus requiring non-steady-state approaches for flux analysis.

Our non-steady-state approach enabled a quantitative analysis of fractional contribution of relevant pathways to the synthesis of UDP-GlcNAc, by partitioning the UDP-GlcNAc molecule into four biochemical modules (glucose, ribose, uracil, and acetyl unit), each of which can utilize glucose as the carbon source. Our analysis indicated a rapid incorporation of labeled ribose via the pentose phosphate pathway and direct glucose incorporation. Slower incorporation occurred for labeled acetyl units and uracil via glycolysis and the Krebs cycle.

Such quantitative deconvolution of UDP-GlcNAc isotopologues into fractional contribution of individual pathways (see Figure 7b) sets the stage for a detailed flux analysis of UDP-GlcNAc biosynthesis and utilization, which is work in progress. Since the FT-ICR-MS and NMR data also provided labeling patterns of numerous other metabolites in the nucleotide sugar pathways, the flux analysis can be readily extended to the network of other nucleotide sugars. Because modular biosynthetic processes occur commonly in cellular metabolism (for example, phospholipid biosynthesis [5]), the approach described should be of general applicability.

## Methods

### Materials

Nucleotide standards were purchased from Sigma Aldrich (St Louis, MO, USA) and used without further purification. All other reagents were of the highest grade commercially available.

### Cell culture

The LnCaP-LN3 prostate cancer cell line was a gift of Dr Clement Ip at the Roswell Park Cancer Institute (Buffalo, NY, USA). Cells were grown in RPMI 1640 medium (Sigma Aldrich, St Louis, MO, USA) supplemented with 10% fetal bovine serum (FBS)(Atlanta Biologicals, Lawrenceville, GA), 100 units/ml penicillin, 100 μg/ml streptomycin, and 0.2% glucose at 37°C and 5% CO_2_. For timecourse experiments, cultures were grown to approximately 70% confluence before replacing medium (20 ml per plate) with 0.2% [U-^13^C] glucose (Sigma Isotec, St. Louis, MO, USA) supplemented RPMI 1640. Initial cell densities were 1.4 × 10^6 ^per plate. The doubling time of the cells under these conditions was approximately 40 h. For timecourse sampling, culture medium was collected and frozen before cells were detached with 0.25% trypsin. After 5-7 min of incubation, trypsin was inactivated with fresh medium, and cells were collected by centrifugation at 1,200 rpm (281 *g*) at 4°C for 5 min. The resulting pellet was resuspended in ice-cold phosphate buffered saline (PBS) for cell counting. Cells were then centrifuged and resuspended again with ice-cold PBS before centrifugation at 4,000 rpm (1,700 *g*) at 4°C for 5 min. The supernatant was removed and the wet cell mass was measured before freezing in liquid N_2 _and subsequent lyophilization. Cells were harvested in duplicates at 0, 3, 6, 11, 24, 34, and 48 h of [U-^13^C] glucose incubation to generate a timecourse of labeling of intracellular metabolites. The corresponding medium samples were analyzed to assess the consumption of glucose and excretion of lactate derived from labeled glucose, as previously described [9,11].

### Polar metabolite extraction

Medium aliquots of 100 μl were extracted with 10% trichloroacetic acid (TCA) and centrifuged at 14,000 rpm for 20 min at 4°C to remove denatured proteins. Supernatant was collected and lyophilized for NMR analysis. The dried medium extract was dissolved in 650 μl D_2_O + 50 nmol 2,2-dimethyl-2-silapentane-5-sulfonic acid-d_6 _(DSS-d_6_) (Sigma Isotec, St. Louis, MO) and transferred to a 5 mm NMR tube. The DSS-d_6 _was used as a standard both for chemical shift and concentration. Dried cell samples were homogenized in 60% CH_3_CN at a 40:1 CH_3_CN:mg dry cell mass ratio and incubated at -80°C for 30 min to promote precipitation of denatured proteins. Samples were then thawed and centrifuged at 14,000 rpm for 20 min at 4°C. Supernatant was collected, and the pellet was washed with 60% CH_3_CN and centrifuged as above. Extracts were combined and lyophilized. Dried cell extracts were dissolved in 350 μl D_2_O + 30 nmol DSS-d_6_, and transferred to 5 mm Shigemi NMR tubes.

### NMR analysis

NMR spectra were recorded at 18.8 T or 14.1 T on Varian Inova spectrometers. All samples were allowed to equilibrate to 20°C inside the magnet before data acquisition. One-dimensional ^1^H experiments were acquired using a standard PRESAT water suppression sequence with a determined 90° pulse, acquisition time of 2 s, and recycle delay of 3 s. Cell and media sample spectra were acquired with 512 and 256 transients, respectively. Two-dimensional TOCSY, heteronuclear single quantum coherence (HSQC), and HSQC-TOCSY NMR experiments were also recorded on selected samples for spectral assignment. TOCSY spectra were generated from a standard pulse sequence with a mixing time of 50 ms, an acquisition time of 341 ms, 56 transients, and 256 increments. HSQC-TOCSY spectra were generated with an acquisition time of 150 ms, 40 transients, and 256 increments. For UDP-GlcNAc identification, commercially available nucleotide sugar standards were prepared in a deuteriated 25 mM K_2_HPO_4 _solution and analyzed by the same set of NMR experiments for comparison with LN3 cell extracts.

### FT-ICR-MS analysis

Following NMR analysis, cell extracts were quantitatively transferred from Shigemi tubes to 2 ml microfuge tubes and lyophilized to remove D_2_O. Samples were then redissolved in H_2_O, diluted to 99% MeOH, and analyzed via direct infusion nanoelectrospray FT-ICR-MS in negative ion mode using a Thermo 7T LTQ FT-ICR-MS, mostly as previously described [5]. The salient conditions were: Advion Nanomate 'A' chip set at -2.1 kV with zero PSI head pressure, resulting in a flow rate of approximately 300 nl/min, and the ICR set to attain a resolution of 200,000 (at m/z 400, 10% valley definition). Exact masses were calculated for UDP-GlcNAc and its isotopologues and assigned using the program PREMISE (Figure 2) as previously described [5]. In order to determine percentage ^13^C enrichment, the intensity of each isotopologue was measured and normalized to the sum of all intensities, that is, to provide the mole fraction of each isotopologue according to Eq. 5:(5)

The intensities were corrected for the contribution from natural abundance using the approach previously described [5].

### Metabolite and positional isotopomer analysis

Metabolite concentrations were determined from ^1^H NMR peak areas of interest using NUTS software (Acorn NMR Inc., Livermore, CA, USA) or Varian integration routines and calibrated against the known concentration of internal standard, DSS-d_6_. For cell extracts, metabolite concentrations were normalized to dry cell mass while medium metabolite concentrations were reported on per ml basis. To correct for differential relaxation, an inversion recovery experiment using an array of delay times from 0 to 10 s was performed on both medium and cell extracts. Saturation factors were then calculated and applied to peak areas based on T_1 _values in order to determine the true intensity as previously described [8,11].

Positional ^13^C enrichment for metabolites was determined by integration of appropriate central and ^13^C satellite peaks in one-dimensional and two-dimensional TOCSY experiments, followed by corrections for differential relaxation where necessary as previously described [8,11].

## Authors' contributions

HNBM developed the GAIMS algorithm and analyzed the MS data; ANL designed and analyzed the NMR and MS data; AB carried out the cell culture experiments and FT-ICR-MS measurement; RMH developed and supervised the MS experiments and developed the PREMISE algorithm; TW-MF designed the cell culture experiments and analyzed data. ANL, HNBM and TW-MF interpreted the data and wrote the manuscript. All authors read and approved the final manuscript.

## Supplementary Material

Additional file 1 **Supplemental figures and table**. Figure S1: metabolite quantification in the medium: consumption and excretion. Figure S2: ^1^H-^13^C heteronuclear single quantum coherence (HSQC)-total correlation spectroscopy (TOCSY) of LN3 cells. Table S1: list of models of ^13^C incorporation into the biochemical units of UDP-*N*-acetyl-D-glucosamine (UDP-GlcNAc). Click here for file
