# A novel deconvolution method for modeling UDP-*N*-acetyl-D-glucosamine biosynthetic pathways based on ^13^C mass isotopologue profiles under non-steady-state conditions

**DOI:** 10.1186/1741-7007-10-74

**Published:** 2012-08-17

**Authors:** Hunter NB Moseley, Andrew N Lane, Alex C Belshoff, Richard M Higashi, Teresa WM Fan

**Affiliations:** 1 Department of Chemistry and Center for Regulatory & Environmental Analytical Metabolomics (CREAM), University of Louisville, Louisville, KY 40292, USA; 2 Structural Biology Program, JG Brown Cancer Center, University of Louisville, Louisville, KY 40292, USA; 3 Department of Medicine, Clinical Translational Research Building, Louisville KY 40202, USA; 4 Department of Pharmacology and Toxicology, University of Louisville, Louisville, KY 40202, USA

## 

The figure published as Figure 2 in the original published version of the manuscript is in fact a duplicate of Figure 5. The correct Figure 2 is shown here (Figure 1 in this correction). Note that the legend for Figure 2 and references to it in the main text apply to the correct Figure 2. The authors and publisher regret the error.

**Figure 1:**
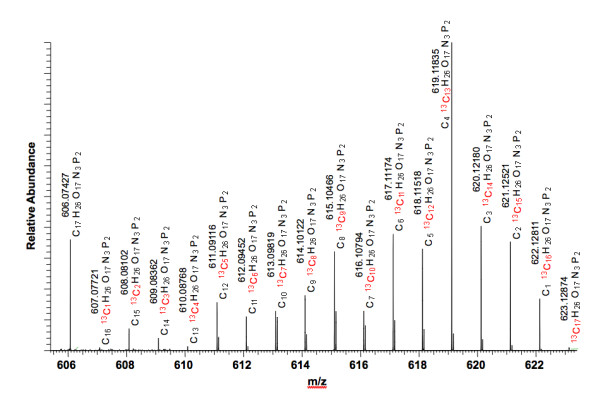
**Species assignments of UDP-*N*-acetyl-D-glucosamine (UDP-GlcNAc) isotopologues in Fourier transform-ion cyclotron resonance-mass spectrometry (FT-ICR-MS)**. The same crude extracts used for NMR were analyzed following re-exchange of ^2^H back to ^1^H. Analysis conditions are stated in the text. With correction to an internal reference, all of the isotopologues were assignable at better than 1 ppm mass accuracy, with most better than 10 ppb mass accuracy. The molecular formulae were assigned using Xcalibur software with elemental limits set to CHONP and allowing up to 17 occurrences of ^13^C. The combination of the ultra-high resolution with extreme mass accuracy resulted in high confidence that only 'pure' ^13^C isotopologues were quantified for the moiety modeling.
