# Supplementary material for: A novel deconvolution method for modeling UDP-N-acetyl-D-glucosamine biosynthetic pathways based on 13C mass isotopologue profiles under non-steady-state conditions
Source: BMC Biol. 2011 May 31;9:37. doi: 10.1186/1741-7007-9-37 (PMC3126751; doi:10.1186/1741-7007-9-37)
Supplement: Additional file 1 — Supplemental figures and table. Figure S1: metabolite quantification in the medium: consumption and excretion. Figure S2: 1H-13C heteronuclear single quantum coherence (HSQC)-total correlation spectroscopy (TOCSY) of LN3 cells. Table S1: list of models of 13C incorporation into the biochemical units of UDP-N-acetyl-D-glucosamine (UDP-GlcNAc). [file 1741-7007-9-37-S1.DOC]

**Supplementary Information for**

**Non-Steady State Modeling of UDP-GlcNAc Biosynthesis Enabled by Stable Isotope Resolved Metabolomics**

Hunter N. B. Moseley, Andrew N. Lane, Alex C. Belshoff, Richard M. Higashi, Teresa W-M. Fan

**Table of Contents**

**Figure S1**. Metabolite quantification in the medium: consumption and excretion.

**Figure S2**. ^1^H-^13^C HSQC-TOCSY of LN3 cells.

**Table S1**. List of models of ^13^C incorporation into the biochemical units of UDP-GlcNAc

**Figure S1. Metabolite quantification in the medium: consumption and excretion.**

The concentrations of metabolites in the medium were determined at different time points by NMR as previously described . ^13^C incorporation and glucose to lactate conversion was calculated from peak areas as previously described . Time course changes were analyzed by linear regression. Over the 48 hr period, the concentration of valine (Val), threonine and leucine in the medium changed insignificantly, whereas the concentration of ^13^C lactate (13CLac) increased and glucose (Gluc) and glutamine (Gln) decreased essentially linearly. Cells were seeded at 1.4x 10^6^ per plate in 20 ml medium. The cell doubling time was approx. 40 h under these conditions. This implies a non-constant rate of nutrient consumption and lactate production as the cell number increased, but the metabolite changed linearly as we have described elsewhere .

From the slopes of Fig. S1, the rate of depletion of ^13^C glucose was 1.39 μmol/h, whereas the consumption of glutamine was 0.19 μmol/h (7-fold less). ^13^C lactate was produced at a rate 1.04 μmol/h, accounting for 38% of the glucose consumption. Based on the cell dry weight measurement at each time point, the initial rate of glucose consumption was calculated to be 1 μmol/h/mg, the rate of glutamine consumption was 0.14 μmol/h/mg and the rate of ^13^C lactate production was 0.74 μmol/h/mg. ^12^C lactate (12CLac) was produced at a rate 10-fold lower than ^13^C lactate. By 48 h approximately 90% of the extracellular lactate was uniformly ^13^C labeled indicating that most, but not all of the newly synthesized lactate was derived from glucose, and about 10% derived from non-glucose sources. Furthermore, ca. 30% of the glucose (Gluc) had been consumed by 48 h.


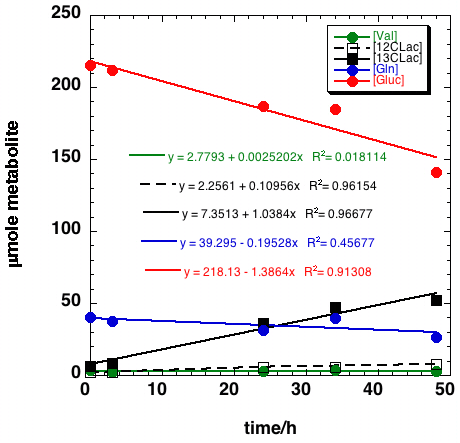


**Figure S2. ^1^H-^13^C HSQC-TOCSY of LN3 cells.**


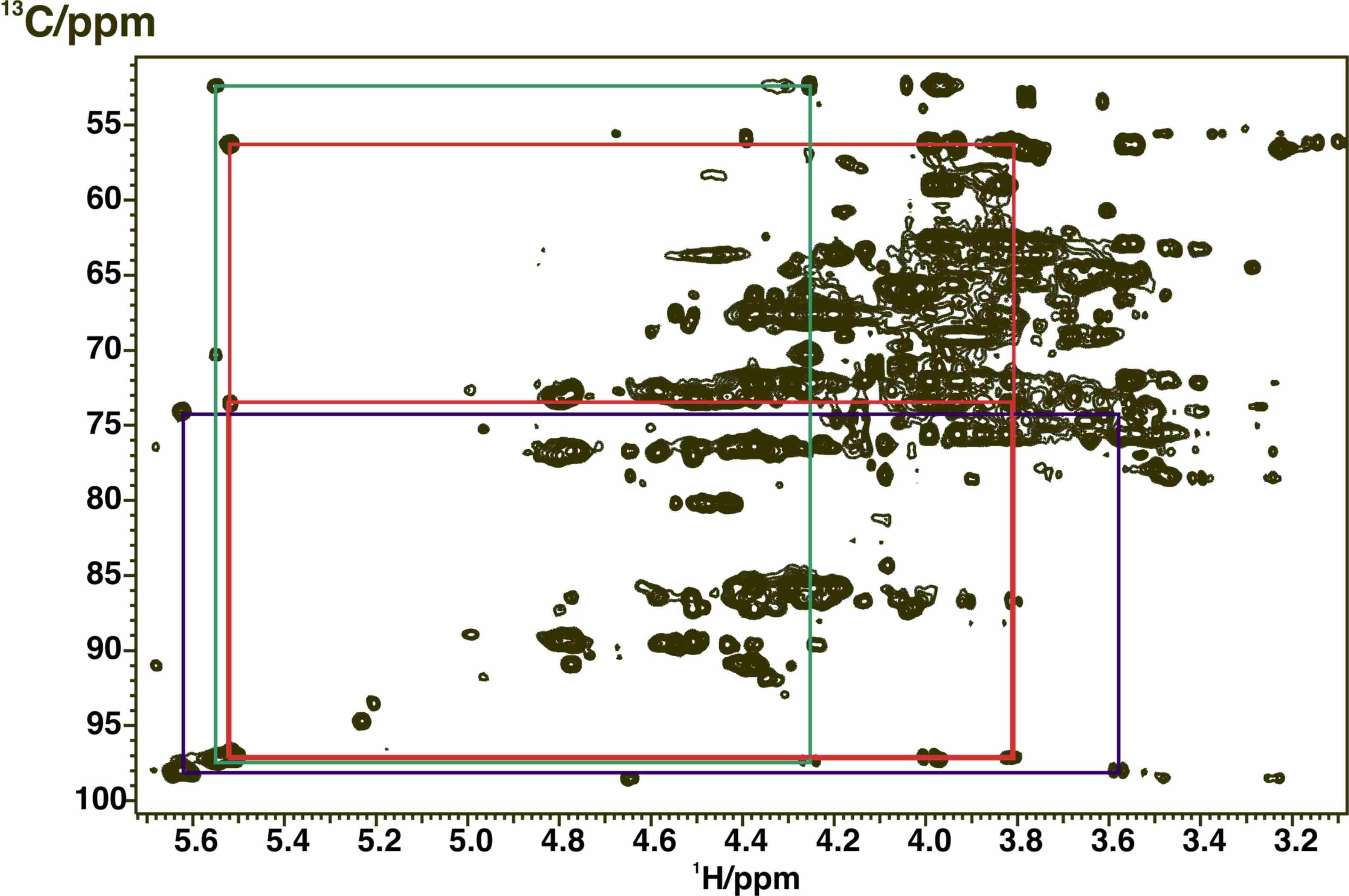
A ^1^H-^13^C HSQC-TOCSY experiment was recorded at 14.1 T on the cell extract at 48 h of growth in [U-^13^C]-glucose. The 2D contour plot is shown. The isotropic mixing time was 50 ms at a B_1_ field strength of 8 kHz. The boxes trace proton-carbon connectivities in the sugar moieties of three UDP-hexoses. Purple is UDP-Glc, red is UDP-GlcNAc and green is UDP-GalNAc.

**Table S1**. List of models of ^13^C incorporation into the biochemical units of UDP-GlcNAc.

Model 1 is the original model as discussed in the text. The remaining 40 models are variants that allow for the presence of different isotopomers of the subunits due to metabolic scrambling. These variants were analyzed in the same way, and the Aikaike criterion was used to find the best models, as described in detail previously .

| **Model #** | **# Parameters** | **Model Description** |
| --- | --- | --- |
| 1 | 6 | g0+g6=1 ; r0+r5=1 ; a0+a2=1 ; u0+u1+u2+u3=1 |
| 2 | 6 | g0+g6=1 ; r0+r5=1 ; a0+a1=1 ; u0+u1+u2+u3=1 |
| 3 | 6 | g0+g5=1 ; r0+r5=1 ; a0+a2=1 ; u0+u1+u2+u3=1 |
| 4 | 6 | g0+g6=1 ; r0+r4=1 ; a0+a2=1 ; u0+u1+u2+u3=1 |
| 5 | 6 | g0+g6=1 ; r0+r5=1 ; a0+a2=1 ; u0+u1+u2+u4=1 |
| 6 | 6 | g0+g3+g6=1 ; r0+r2+r3+r5=1 ; a0+a2=1 ; u0+u1+u2+u3=1 ; g3=r2*2 ;  r3=r2 ; g0=r0 ; g6=r5 |
| 7 | 7 | g0+g6=1 ; r0+r5=1 ; a0+a1+a2=1 ; u0+u1+u2+u3=1 |
| 8 | 7 | g0+g6=1 ; r0+r5=1 ; a0+a2=1 ; u0+u1+u2+u3=1 ; ^a^c0+c1=1 |
| 9 | 7 | g0+g6=1 ; r0+r5=1 ; a0+a2=1 ; u0+u1+u2+u3+u4=1 |
| 10 | 7 | g0+g1+g6=1 ; r0+r5=1 ; a0+a2=1 ; u0+u1+u2+u3=1 |
| 11 | 7 | g0+g2+g6=1 ; r0+r5=1 ; a0+a2=1 ; u0+u1+u2+u3=1 |
| 12 | 7 | g0+g3+g6=1 ; r0+r5=1 ; a0+a2=1 ; u0+u1+u2+u3=1 |
| 13 | 7 | g0+g4+g6=1 ; r0+r5=1 ; a0+a2=1 ; u0+u1+u2+u3=1 |
| 14 | 7 | g0+g5+g6=1 ; r0+r5=1 ; a0+a2=1 ; u0+u1+u2+u3=1 |
| 15 | 7 | g0+g6=1 ; r0+r1+r5=1 ; a0+a2=1 ; u0+u1+u2+u3=1 |
| 16 | 7 | g0+g6=1 ; r0+r2+r5=1 ; a0+a2=1 ; u0+u1+u2+u3=1 |
| 17 | 7 | g0+g6=1 ; r0+r3+r5=1 ; a0+a2=1 ; u0+u1+u2+u3=1 |
| 18 | 7 | g0+g6=1 ; r0+r4+r5=1 ; a0+a2=1 ; u0+u1+u2+u3=1 |
| 19 | 7 | g0+g3+g6=1 ; r0+r2+r3+r5=1 ; a0+a2=1 ; u0+u1+u2+u3=1 ; g3=r2*2 ;  r3=r2 |
| 20 | 7 | g0+g3+g6=1 ; r0+r2+r3+r5=1 ; a0+a1+a2=1 ; u0+u1+u2+u3=1 ; g3=r2*2 ;  r3=r2 ; g0=r0 ; g6=r5 |
| 21 | 7 | g0+g3+g6=1 ; r0+r2+r3+r4+r5=1 ; a0+a2=1 ; u0+u1+u2+u3=1 ; g3=r2*2 ;  r3=r2 ; g0=r0 ; g6=r5 |
| 22 | 7 | g0+g3+g5+g6=1 ; r0+r2+r3+r4+r5=1 ; a0+a2=1 ; u0+u1+u2+u3=1 ; g3=r2*2 ;  r3=r2 ; g0=r0 ; g6=r5 ; g5=r4 |
| 23 | 8 | g0+g6=1 ; r0+r5=1 ; a0+a1+a2=1 ; u0+u1+u2+u3=1 ; ^a^c0+c1=1 |
| 24 | 8 | g0+g1+g6=1 ; r0+r5=1 ; a0+a1+a2=1 ; u0+u1+u2+u3=1 |
| 25 | 8 | g0+g2+g6=1 ; r0+r5=1 ; a0+a1+a2=1 ; u0+u1+u2+u3=1 |
| 26 | 8 | g0+g3+g6=1 ; r0+r5=1 ; a0+a1+a2=1 ; u0+u1+u2+u3=1 |
| 27 | 8 | g0+g4+g6=1 ; r0+r5=1 ; a0+a1+a2=1 ; u0+u1+u2+u3=1 |
| 28 | 8 | g0+g5+g6=1 ; r0+r5=1 ; a0+a1+a2=1 ; u0+u1+u2+u3=1 |
| 29 | 8 | g0+g6=1 ; r0+r1+r5=1 ; a0+a1+a2=1 ; u0+u1+u2+u3=1 |
| 30 | 8 | g0+g6=1 ; r0+r2+r5=1 ; a0+a1+a2=1 ; u0+u1+u2+u3=1 |
| 31 | 8 | g0+g6=1 ; r0+r3+r5=1 ; a0+a1+a2=1 ; u0+u1+u2+u3=1 |
| 32 | 8 | g0+g6=1 ; r0+r4+r5=1 ; a0+a1+a2=1 ; u0+u1+u2+u3=1 |
| 33 | 8 | g0+g3+g6=1 ; r0+r2+r3+r5=1 ; a0+a1+a2=1 ; u0+u1+u2+u3=1 ; g3=r2*2 ;  r3=r2 |
| 34 | 8 | g0+g6=1 ; r0+r2+r3+r5=1 ; a0+a1+a2=1 ; u0+u1+u2+u3=1 ; r3=r2 |
| 35 | 8 | g0+g3+g5+g6=1 ; r0+r2+r3+r5=1 ; a0+a1+a2=1 ; u0+u1+u2+u3=1 ; g3=r2*2 ;  r3=r2 ; g6=r5 |
| 36 | 9 | g0+g1+g6=1 ; r0+r2+r3+r5=1 ; a0+a1+a2=1 ; u0+u1+u2+u3=1 ; r3=r2 |
| 37 | 9 | g0+g2+g6=1 ; r0+r2+r3+r5=1 ; a0+a1+a2=1 ; u0+u1+u2+u3=1 ; r3=r2 |
| 38 | 9 | g0+g3+g6=1 ; r0+r2+r3+r5=1 ; a0+a1+a2=1 ; u0+u1+u2+u3=1 ; r3=r2 |
| 39 | 9 | g0+g4+g6=1 ; r0+r2+r3+r5=1 ; a0+a1+a2=1 ; u0+u1+u2+u3=1 ; r3=r2 |
| 40 | 9 | g0+g5+g6=1 ; r0+r2+r3+r5=1 ; a0+a1+a2=1 ; u0+u1+u2+u3=1 ; r3=r2 |
| 41 | 9 | g0+g3+g5+g6=1 ; r0+r2+r3+r5=1 ; a0+a1+a2=1 ; u0+u1+u2+u3=1 ; r3=r2 ;  g6=r5 |

^a^Refers to the carbon in uracil which is derived from CO_2_.

**References**

1. Fan TW, Lane AN: **Structure-based profiling of Metabolites and Isotopomers by NMR.** *Progress in NMR Spectroscopy* 2008, **52**:69-117

2. Fan TW-M, Kucia M., Jankowski K, Higashi RM, Rataczjak MZ, Rataczjak J, Lane AN: **Proliferating Rhabdomyosarcoma cells shows an energy producing anabolic metabolic phenotype compared with Primary Myocytes.** *Molecular Cancer* 2008, **7**:79.

3. Lane AN, Fan TW: **Quantification and identification of isotopomer distributions of metabolites in crude cell extracts using ^1^H TOCSY**. *Metabolomics* 2007, **3**:79-86.

4. Lane AN, Fan TW, Higashi RM: **Isotopomer-based metabolomic analysis by NMR and mass spectrometry.** *Biophysical Tools for Biologists* 2008, **84**:541-588.

5. Lane AN, Fan TW-M, Bousamra II M, Higashi RM, Yan J, Miller DM: **Stable Isotope-Resolved Metabolomics (SIRM) in Cancer Research with Clinical Application to Non-Small Cell Lung Cancer**. *Omics* 2011, **15**:173-182.

6. Akaike H: **A new look at the statistical model identification.** *IEEE Transactions on Automatic Control* 1974, **19**:716–723

7. Moseley HNB, Higashi RM, Fan TW-M, Lane AN: **Analysis of Non-Steady State Stable Isotope-Resolve Metabolism of UDP-GlcNAc and UDP-GalNAc.** *Proceedings of Bioinformatics 2011* 2011, **in press**.
